# Positioning Accuracy Determination of the Servo Axes for Grinding Wavy-Tilt-Dam Seals Using a Four-Axis Grinder

**DOI:** 10.3390/mi12040388

**Published:** 2021-04-02

**Authors:** Guang Feng, Xiaobao Ma

**Affiliations:** 1 College of Mechanical and Vehicle Engineering, Taiyuan University of Technology, Taiyuan 030024, China; maxiaobao@tyut.edu.cn; 2 Engineering Research Center of Advanced Metal Composites Forming Technology and Equipment, Ministry of Education, Taiyuan 030024, China; 3 TYUT-UOW Joint Research Centre of Advanced Forming and Manufacturing Technology, Taiyuan 030024, China; 4 Taiyuan Tongze Heavy Industry Co., Ltd., Taiyuan 030032, China

**Keywords:** WTD seal, wavy face, grinding, positioning accuracy, form error

## Abstract

The wavy-tilt-dam (WTD) seal is considered to be one of the ideal sealing patterns used in nuclear reactor coolant pumps (RCPs). Grinding such seals with a four-axis grinder had been proposed and six grinding implementation strategies were described in our previous studies. However, another important issue is to determine the positioning accuracy of each servo axis so that the high-precision moving components can be selected properly. In the present paper, the positioning accuracy analysis is carried out to seek a balance between the manufacturing cost and the accuracy requirements. First, a geometric model is established for investigating the error sensitivity of each axis and setting reasonable accuracy allocation of the four axes. Subsequently, the combined influence of all four axes is assessed based on multi-body system (MBS) theory and homogeneous transformation matrix (HTM). According to the results calculated, positioning errors of the *X*-axis, *Z*-axis, *B*-axis, and *C*-axis within ±10 μm, ±0.1 μm, ±1 arcsec and ±60 arcsec are acceptable, respectively. Meanwhile, the form error calculated of the ground wavy face is no more than 109.74 nm. It is indicated that the accuracy level of the moving components is achievable by modern manufacturing techniques. The present paper is expected to serve as a theoretical basis for the design and development of the four-axis grinder.

## 1. Introduction

The wavy-tilt-dam (WTD) seal is one of the hydrodynamic mechanical face seals used in nuclear reactor coolant pumps (RCPs) [1]. Generally, one RCP is equipped with three WTD seals, and each seal has the ability to undertake an entire system load of no less than 15.3 MPa [2,3]. Therefore, it is desirable that the WTD seals possess excellent mechanical properties, heat transfer characteristics, thermal environment resistance, chemical stability, sliding compatibility, and high durability [4,5,6]. Those seals are usually made of hard and brittle ceramic materials, such as alumina oxide, silicon carbide, and silicon nitride or tungsten carbide hard alloy, which make it difficult to achieve mechanical machining [7,8]. Meanwhile, the WTD seals are typically large-sized parts whose diameter can reach 300 mm, while the maximal wave amplitude is only several micrometers. The form error should be within the length of two helium light bands (0.58 μm) and the surface roughness should be at the nanometric scale to avoid surface/subsurface damages [9,10,11]. Overall, forming such a wavy face with extremely high manufacturing requirements using existing methods—for instance, lapping, polishing, general grinding, laser manufacturing, etc.—will inevitably encounter various inherent defects [12,13,14,15,16,17]. As a solution, a high accuracy line-contact envelope grinding method with a large diamond cup wheel based on a four-axis grinder was elaborated to fabricate such seals, and the detailed analyses of the forming principle were discussed in our previous paper [18]. Noticeably, since the form error of a ground seal surface is extremely sensitive to the different linkage relations of the four axes; a study was presented, and six implementation strategies were given to evaluate the available principle form errors. It was found that the minimal principle form error was only 9.64 nm and the maximum was 57.88 [19]. These results imply that grinding with a four-axis ultraprecision grinder can provide an accessible approach to manufacturing the wavy face and that there are several different implementation strategies that can be selected.

However, another key issue that is of high concern when designing and developing the proposed four-axis grinder is determining the positioning accuracy so as to choose the servo axes economically. Generally, there are many factors that can affect the machining accuracy including geometric errors, kinematic errors, thermally induced errors, cutting force-induced errors, servo errors, fixture-dependent errors, etc. Geometric error is one of the primary error sources in multi-axis machine tools and it is a significant criterion for machine tool manufacturers when assessing accuracy levels [20,21]. Hence, it is necessary to evaluate the impact of geometric errors generated by each axis (and even the form error of the workpiece) on the accuracy of a machine tool to find the vital errors that need to be identified and which one should be concerned about [22]. In order to discuss the effects of the moving component errors on the position of the grinding wheel relative to the workpiece, their spatial relationship must be defined and clarified. The generalized model used to evaluate geometric errors is normally based on a multi-body system (MBS), theory, and a homogeneous transformation matrix (HTM). Zhong et al. [23] established a five-axis machining center prototype as an MBS and exhibited a position geometric error modeling, identification, and compensation method. Fu et al. [24] established a differential motion matrix of each axis based on HTM and presented the precision enhancement of five-axis machine tools. Lee et al. [25] demonstrated a method to identify and measure the position-independent geometric errors of a five-axis machine tool based on the MBS model. Zhu et al. [26] reported a five-axis machine tool model based on the kinematics of MBS and 4 × 4 HTM to present a general geometric error calculation method. Wu et al. [27] established relative motion constraint equations about the tooltip position and tool orientation vector relative to the referred five-axis machine tool based on HTM and MBS theory and discussed a new compensation methodology of geometric errors to improve the machining accuracy. Guo et al. [28] proposed a methodology to analyze and compensate for the effect of geometric errors in order to improve the precision of a five-axis machine tool with MBS theory and HTM. Apparently, geometric error analysis can establish a relationship between the positioning accuracy of the servo axes and the form accuracy of the workpiece.

In the present paper, the basic model for grinding the WTD seals is established according to the first strategy mentioned in our previous study [19] which produces the maximal form error. Obviously, if motional accuracy of the axes is achieved on basis of this strategy, fulfilling the machining requirements of the WTD seals, then other implementation strategies are all undoubtedly feasible. Meanwhile, the main function of each axis is analyzed separately and their influence on the ground form error of the wavy seal face is calculated with an assumed positioning error. Subsequently, combined with product technical parameters provided by famous companies who fabricate ultra-precision machine tools, the positioning accuracy of each axis will be determined preliminarily. Eventually, these geometric error components are transformed to the coordinate frame of the four-axis grinder based on MBS theory, and the integrated influence of the ground form error of the WTD seal is presented by comparing it with the design value. The results will be an important reference for choosing moving components economically and in accordance with modern manufacturing techniques. This study is expected to serve as the theoretical basis for the design and development of the proposed four-axis grinder.

## 2. The Modelling of a Wavy Face Seal by a Four-Axis Grinder Using a Cup Wheel

The equation of the WTD seal in a cylindrical coordinate system is

(1) $$\{\begin{array}{l} x=R\cos\theta \\ y=R\sin\theta \\ z=\{\begin{array}{l} 0;\\ \gamma_{0}(R-R_{2})(\cos(N\theta )-1); \end{array}\begin{array}{c} R_{1}\leq R\leq R_{2}\\ R_{2}\leq R\leq R_{3} \end{array} \end{array}$$

where *R* is the radius of a circle in which an arbitrary point is located, *R*_1_ is the inner radius of the seal face, *R*_2_ is the inner radius of the wavy face, *R*_3_ is the outer radius of the seal face, *γ*_0_ is the proportionality coefficient of the radial profile tilt angle, *N* is the number of waves and *θ* is the phase angle.

**Figure 1:**
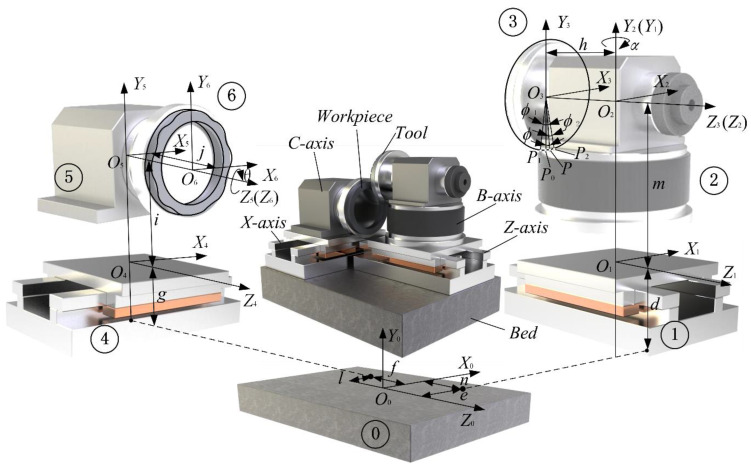
Coordinate analysis diagram for WTD seal forming based on a four-axis grinder.

The coordinate analysis diagram for WTD seal forming based on a four-axis grinder is shown in Figure 1. Point *O*_3_ is designated as the origin of the cup wheel coordinate system *O*_3_-*X*_3_*Y*_3_*Z*_3_. The *Z*_3_-axis is consistent with the wheel axis. The *Y*_3_-axis passes through point *P*_0_ which is the lowest point of the cup wheel. *P*_1_ and *P*_2_ are the start point and endpoint of the grinding contact arc, whose phase angles are denoted by *ϕ*_1_ and *ϕ*_2_, respectively. Point *O*_2_ is designated as the origin of the rotary table coordinate system *O*_2_-*X*_2_*Y*_2_*Z*_2_ that is obtained by translating *h* from coordinate system *O*_3_-*X*_3_*Y*_3_*Z*_3_ along the *Z*_3_-axis. The radical profile tilt angle of the wavy face is obtained by swinging *α* around the *Y*_2_-axis from coordinate system *O*_2_-*X*_2_*Y*_2_*Z*_2_ to coordinate system *O*_1_-*X*_1_*Y*_1_*Z*_1_. Coordinate system *O*_1_-*X*_1_*Y*_1_*Z*_1_ is located on the *Z*-axis slide carriage that is obtained by translating *m* from coordinate system *O*_2_-*X*_2_*Y*_2_*Z*_2_ along *Y*_2_-axis. After translating *e*, *d*, *n*, the machine coordinate system *O*_0_-*X*_0_*Y*_0_*Z*_0_ is determined. Similarly, coordinate system *O*_4_-*X*_4_*Y*_4_*Z*_4_ of the *X*-axis slide carriage is obtained by translating *l*, *g*, *f* from coordinate system *O*_0_-*X*_0_*Y*_0_*Z*_0_, while coordinate system *O*_5_-*X*_5_*Y*_5_*Z*_5_ is formed by translating *i* from coordinate system *O*_4_-*X*_4_*Y*_4_*Z*_4_ along *Y*_5_-axis. *C*-axis coordinate system *O*_6_-*X*_6_*Y*_6_*Z*_6_ is achieved by translating *j* from coordinate system *O*_5_-*X*_5_*Y*_5_*Z*_5_ along *X*_5_-axis. The phase angle of the seal is *θ*.

To describe the generation of the wavy face, an arbitrary point *P* on the contact arc is taken for mathematical calculations. The coordinates of point *P* in coordinate system *O*_3_-*X*_3_*Y*_3_*Z*_3_ are (*R*_w_sin*ϕ*, −*R*_w_cos*ϕ*, 0), where *R*_w_ is the radius of the cup wheel and −*π* ≤ *ϕ* ≤ *π*. The coordinates of point *P* can be written as

(2) $$\left[\begin{array}{c} x_{3}\\ y_{3}\\ z_{3}\\ 1 \end{array}\right]=\left[\begin{array}{c} R_{w}\sin\phi \\ -R_{w}\cos\phi \\ 0\\ 1 \end{array}\right]$$

The coordinates of point *P* in coordinate system *O*_2_-*X*_2_*Y*_2_*Z*_2_ are

(3) $$\left[\begin{array}{c} x_{2}\\ y_{2}\\ z_{2}\\ 1 \end{array}\right]=\left[\begin{array}{cccc} 1 & 0 & 0 & 0\\ 0 & 1 & 0 & 0\\ 0 & 0 & 1 & -h\\ 0 & 0 & 0 & 1 \end{array}\right]\left[\begin{array}{c} x_{3}\\ y_{3}\\ z_{3}\\ 1 \end{array}\right]$$

After *B*-axis rotates *α*, the coordinates of point *P* in coordinate system *O*_1_-*X*_1_*Y*_1_*Z*_1_ are

(4) $$\left[\begin{array}{c} x_{1}\\ y_{1}\\ z_{1}\\ 1 \end{array}\right]=\left[\begin{array}{cccc} \cos\alpha & 0 & \sin\alpha & 0\\ 0 & 1 & 0 & m\\ -\sin\alpha & 0 & \cos\alpha & 0\\ 0 & 0 & 0 & 1 \end{array}\right]\left[\begin{array}{c} x_{2}\\ y_{2}\\ z_{2}\\ 1 \end{array}\right]$$

The coordinates of point *P* in coordinate system *O*_0_-*X*_0_*Y*_0_*Z*_0_ are

(5) $$\left[\begin{array}{c} x_{0}\\ y_{0}\\ z_{0}\\ 1 \end{array}\right]=\left[\begin{array}{cccc} 1 & 0 & 0 & e\\ 0 & 1 & 0 & d\\ 0 & 0 & 1 & n\\ 0 & 0 & 0 & 1 \end{array}\right]\left[\begin{array}{c} x_{1}\\ y_{1}\\ z_{1}\\ 1 \end{array}\right]$$

The coordinates of point *P* in coordinate system *O*_4_-*X*_4_*Y*_4_*Z*_4_ are

(6) $$\left[\begin{array}{c} x_{4}\\ y_{4}\\ z_{4}\\ 1 \end{array}\right]=\left[\begin{array}{cccc} 1 & 0 & 0 & l\\ 0 & 1 & 0 & -g\\ 0 & 0 & 1 & f\\ 0 & 0 & 0 & 1 \end{array}\right]\left[\begin{array}{c} x_{0}\\ y_{0}\\ z_{0}\\ 1 \end{array}\right]$$

The coordinates of point *P* in coordinate system *O*_5_-*X*_5_*Y*_5_*Z*_5_ are

(7) $$\left[\begin{array}{c} x_{5}\\ y_{5}\\ z_{5}\\ 1 \end{array}\right]=\left[\begin{array}{cccc} 1 & 0 & 0 & 0\\ 0 & 1 & 0 & -i\\ 0 & 0 & 1 & 0\\ 0 & 0 & 0 & 1 \end{array}\right]\left[\begin{array}{c} x_{4}\\ y_{4}\\ z_{4}\\ 1 \end{array}\right]$$

Finally, the coordinates of point *P* in coordinate system *O*_6_-*X*_6_*Y*_6_*Z*_6_ are

(8) $$\left[\begin{array}{c} x_{6}\\ y_{6}\\ z_{6}\\ 1 \end{array}\right]=\left[\begin{array}{cccc} \cos\theta & -\sin\theta & 0 & 0\\ \sin\theta & \cos\theta & 0 & 0\\ 0 & 0 & 1 & -j\\ 0 & 0 & 0 & 1 \end{array}\right]\left[\begin{array}{c} x_{5}\\ y_{5}\\ z_{5}\\ 1 \end{array}\right]=\left[\begin{array}{c} \left(R_{w}\cos\alpha \sin\phi -h\sin\alpha +e+l\right)\cos\theta -\left(-R_{w}\cos\phi +m+d-g-i\right)\sin\theta \\ \left(R_{w}\cos\alpha \sin\phi -h\sin\alpha +e+l\right)\sin\theta +\left(-R_{w}\cos\phi +m+d-g-i\right)\cos\theta \\ -R_{w}\sin\alpha \sin\phi -h\cos\alpha +n+f-j\\ 1 \end{array}\right]$$

Equation (8) can be rewritten in concise form as

(9) $$\{\begin{array}{c} x_{6}=u(R_{w},d,e,f,g,i,j,l,m,n,h,\alpha ,\theta ,\phi )\\ y_{6}=v(R_{w},d,e,f,g,i,j,l,m,n,h,\alpha ,\theta ,\phi )\\ z_{6}=w(R_{w},d,e,f,g,i,j,l,m,n,h,\alpha ,\theta ,\phi ) \end{array}$$

With the first implementation strategy mentioned in the previous study [19] that proposes *R*_0_ = (*R*_2_ + *R*_3_)/2, the equation of the intersection line between the wavy face and a specified cylindrical surface is given by

(10) $$\left[\begin{array}{c} x_{6}\\ y_{6}\\ z_{6} \end{array}\right]=\left[\begin{array}{c} R_{0}\cos\theta \\ R_{0}\sin\theta \\ \gamma_{0}\left(R_{0}-R_{1}\right)\left(\cos\left(N\right)-1\right) \end{array}\right]$$

Substituting *ϕ* = 0 into Equation (8) to obtain the coordinates of point *P*_0_ and then letting their values equal to those in Equation (10). As a result, *l*, *m* and *n* are given by

(11) $$\{\begin{array}{l} l=R_{0}+h\sin\alpha -e\\ m=R_{w}+g+i-d\\ n=\gamma_{0}\left(R_{0}-R_{2}\right)\left(\cos\left(N\theta \right)-1\right)+h\cos\alpha -n-f+j \end{array}$$

At an arbitrary moment, *α* = *γ*_0_(cos(*Nθ*) − 1). The generated form deviation can be expressed by

(12) $$\{\begin{array}{l} x_{6}=u(R_{w},d,e,f,g,i,j,l,m,n,h,\alpha ,\theta ,\phi )\\ y_{6}=v(R_{w},d,e,f,g,i,j,l,m,n,h,\alpha ,\theta ,\phi )\\ z_{6}=w(R_{w},d,e,f,g,i,j,l,m,n,h,\alpha ,\theta ,\phi )-\gamma_{0}\left(\sqrt{x^{2}+y^{2}}-R_{2}\right)\left(\cos\left(N\arctan\frac{y}{x}\right)-1\right) \end{array}$$

A common WTD seal was taken as an implementation case and its parameters are: *R*_1_ = 130 mm, *R*_2_ = 135 mm, *R*_3_ = 150 mm, *γ*_0_ = 0.0002 rad, *N* = 9. Other parameters are: *R*_w_ = 175 mm, *d* = 120 mm, *e* = 300 mm, *f* = 350 mm, *g* = 120 mm, *i* = 200 mm, *j* = 150 mm, *m* = 175mm, *h* = 200 mm. The principle form error is 57.88 nm.

## 3. Form Error Calculations Concerning Geometric Errors of the Four Axes

Generally, all rigid bodies have three rotational and three translational error components associated with their motion and their body position in the reference frame. Actually, the geometric error of the machine tool is mainly affected by linear and angular positioning errors generated by the individual linear and rotational axes in most cases. Therefore, it is necessary to investigate the functions of each axis and determine the error sensitive direction. Then, the accuracy degree of the moving axis can be obtained accordingly. What will be stressed is that, in the present four-axis grinder, each axis has a specified function. The *X*-axis serves as a linear axis to control the location of the lowest point of the cup wheel in real time. The *Z*-axis is used to accommodate the depth of cut and axial location of the wavy face. Meanwhile, it is also used to control the location of the seal dam. The *B*-axis is a swing axis to generate the wavy face. The *C*-axis can be considered as an indexing rotary table controlling the location of the waves in a circumferential direction. To evaluate the accuracy requirement of each axis, an assumed positioning error will be given to calculate the consequent form error of the wavy face. In addition, a generalized error model used to evaluate the geometric errors based on MBS theory and HTM will be established taking all the axes into considerations.

## 4. The Effects of the Positioning Errors on Form Error of the Ground Wavy Face

Assuming that the positioning errors of *X*-axis, *Z*-axis, *B*-axis and *C*-axis are defined as *δ*_x,_ *δ*_z_, *ε*_α,_ and *ε*_θ_, respectively, the actual location of the four axes are thus:

(13) $$l^{\prime }=R_{0}+h\sin\alpha -e+\delta_{x}$$

(14) $$n^{\prime }=\gamma_{0}\left(R_{0}-R_{2}\right)\left(\cos\left(N\theta \right)-1\right)+h\cos\alpha -n-f+j+\delta_{z}$$

(15) $$\alpha^{\prime }=\alpha +\varepsilon_{\alpha }$$

(16) $$\theta^{\prime }=\theta +\varepsilon_{\theta }$$

Referring to Equation (8), it can be found that the positioning error of the *X*-axis affects the form error of the ground wavy face in the *X*- and *Y*- direction. It is obvious that the value of *n’* just affects the coordinates of point *P* in the *Z* direction which represents the location of the wavy face and the seal dam in the axial direction. It will not affect the form error of the ground wavy face. The actual location of the *B*-axis will influence the form error of the ground wavy face in three directions. Since the direction of the *B*-axis positioning error coincides with the seal waves, its value will affect the ground shape of the waves seriously. The actual location of the *C*-axis will also affect the form error of the ground wavy face in three directions, while the main influence is the location of the waves in tangential direction but not the amplitude of the waves. Thus, the positioning accuracy requirement of the *C*-axis can be degraded slightly.

## 5. Positioning Accuracy Determination of the Servo Axes

To demonstrate the influence of positioning error among different axes on the ground wavy face, a series of assumed values are given to calculate the ground form error according to product catalogues provided by several motional component manufacturers. The positioning errors selected of the transitional and rotational axes are given in column no. 2 of Table 1 and Table 2, respectively. Since the ground principle form error of the dam surface is equal to 0, it will be simplified by taking the dimensions of the inner radius to the outer radius of the wavy face for the form error calculations. Each assumed positioning error of the axis will be considered as an independent variable and substituted into Equation (12). The calculation results are shown in column no. 3 of Table 1 and Table 2. It is obvious that positioning errors within ±10 μm of *X*-axis, ±0.1 μm of *Z*-axis, ±1 arcsec of *B*-axis, ±60 arcsec of *C*-axis, are acceptable.

**Figure 2:**
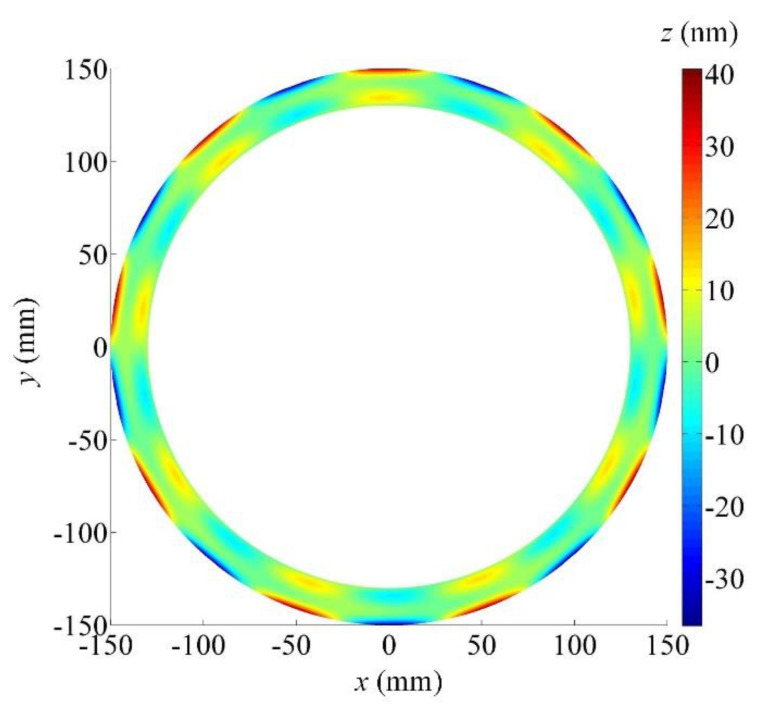
Form deviations map of the ground wavy face concerning the *X*-axis positioning error (10 μm).

When the positioning error of the *X*-axis is equal to 10 μm, the form deviations map of the ground wavy face is shown in Figure 2. It is obvious that the maximum form deviations are generated on the outer edge of the wavy face and the form error is less than 80 nm. Though *X*-axis is a servo-controlled axis, it locates within the insensitive direction of the processing error. The positioning accuracy of such an axis just affects the width of the dam surface. Therefore, the positioning error of the *X*-axis within ±10 μm is sufficient for wavy face grinding.

Since the positioning error of the *Z*-axis just influences the axial position of the wavy face, its value does not affect the ground form error in principle. However, it is recommended that the positioning error of the *Z*-axis should be set to a higher degree. One reason is that it locates within the sensitive direction of the processing error. The other reason is that the boundary between the wavy face and the dam surface is controlled by the motional accuracy of the *Z*-axis. Therefore, its positioning error must be restricted to within 0.5 μm, or even 0.1 as the other factors causing form error in the actual machining process need to be considered.

**Figure 3:**
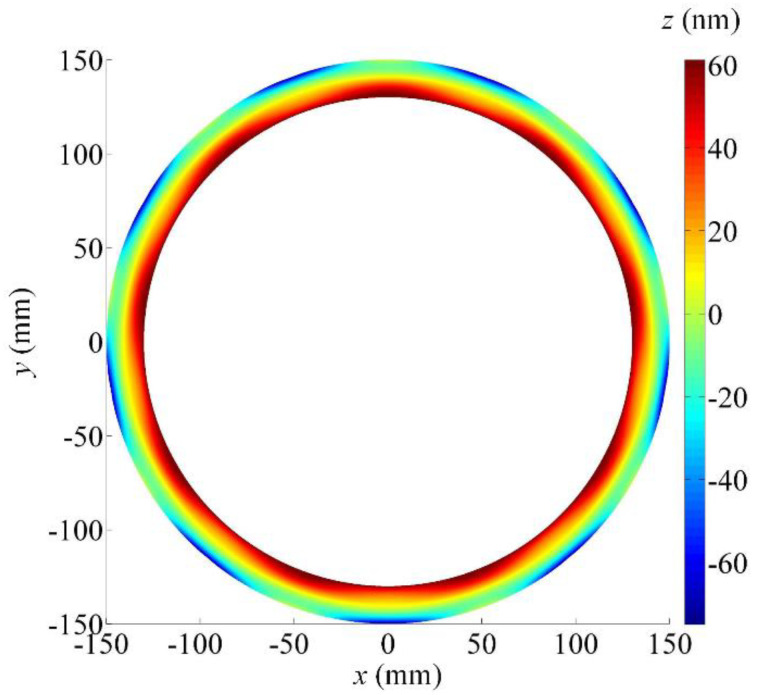
Form deviations map of the ground wavy face concerning the *B*-axis positioning error (1 arcsec).

The form deviations map of the ground wavy face when the positioning error of the *B*-axis is equal to 1 arcsec is shown in Figure 3. The maximum form deviation is generated on both the outer and inner edges of the wavy face and the form error is more than 120 nm. Apparently, the positioning error of the *B*-axis affects the form error of the ground wavy face greatly. The positioning accuracy of the *B*-axis should be high enough.

**Figure 4:**
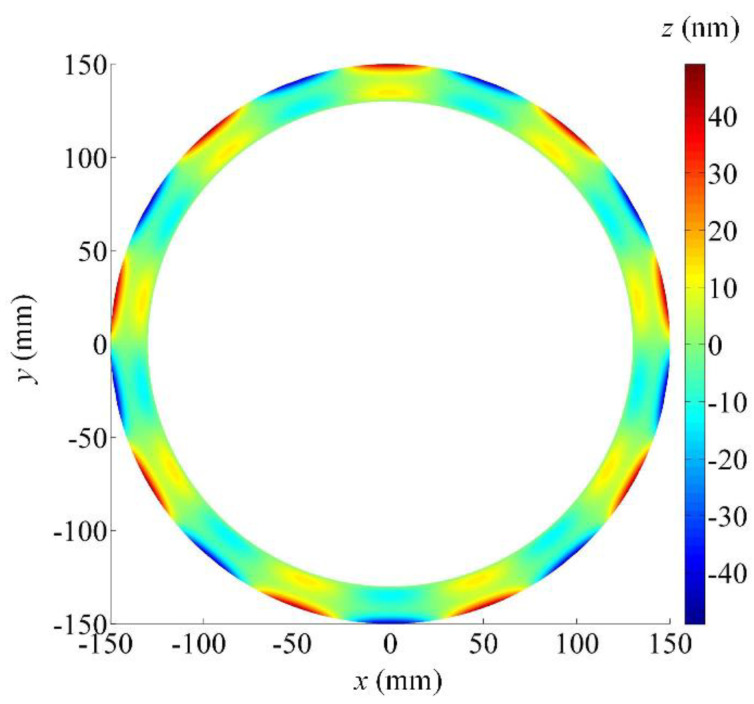
Form deviations map of the ground wavy face concerning the *C*-axis positioning error (60 arcsec).

The form deviations map of the ground wavy face when the positioning error of the *C*-axis is equal to 60 arcsec is shown in Figure 4. The maximum of the form deviations is generated on the outer edge of the deviation map and the form error is more than 90 nm. Compared with the *B*-axis, the positioning accuracy has been expanded by an order of magnitude but the form error decreases. It can be found that the influence of the *C*-axis positioning accuracy on the form error of the wavy face is relatively small.

## 6. Effects of Four-Axis Geometric Errors on Form Error of the Ground Wavy Face

**Figure 5:**
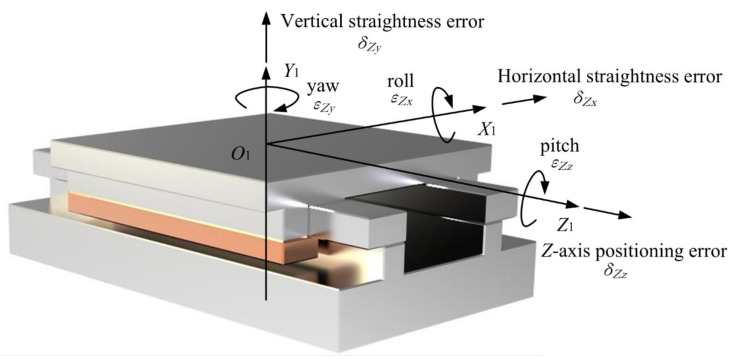
Schematic diagram for the linear and angular error analysis of a translational axis.

**Figure 6:**
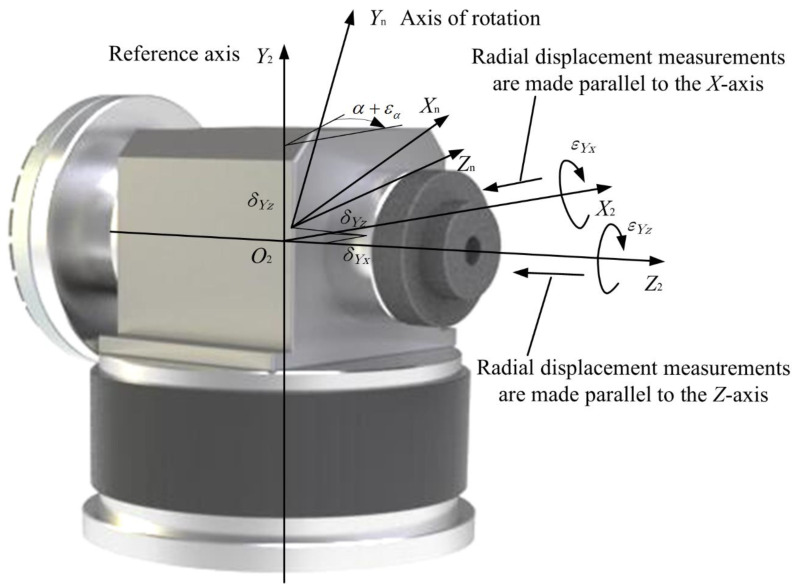
Schematic diagram for a linear and angular error analysis of a rotational axis.

It is known that each axis has six geometric error components. For a translational axis, the error components include one positioning error, two straightness errors, and three angular errors (roll, yaw and pitch). For a rotational axis, the linear errors include one axial error and two radial errors and the angular errors are one angular position error and two tilt errors [29]. The schematic diagrams for a linear and angular error analysis of the translational and rotational axes are shown in Figure 5 and Figure 6.

Generally, the squareness error between the two translational axes should also be considered. Therefore, the four-axis grinder has 25 geometric errors in total. In the present paper, *δ_ij_* represents the linear errors and *θ_ij_* represents angular error, where the first subscript represents the motion axis and the second subscript represents the error direction or the rotation axis of an angular error. Due to the fact that the squareness error associated with the range of motion between the two translational axes is extremely small, this error component is ignored in this case. Assuming the coordinates of point *P* are expressed by *P*_a_, the actual coordinates of point *P* can be described as Equation (17) based on the transformation matrix and by taking the geometric errors into consideration.

(17) $$P_{a}={\left[\begin{array}{cccc} x_{a} & y_{a} & z_{a} & 1 \end{array}\right]}^{T}$$

Its values can be formulated using the following equation:

(18) $$P_{a}=R_{C}T_{X}T_{Z}R_{B}P_{W}$$

where *R*_C_ is the actual rotation matrices of the *C*-axis, *T*_X_ is the actual translation matrices from the *B*-axis to the *X*-axis, *T*_Z_ is the actual translation matrices of the *Z*-axis, *R*_B_ is the actual rotation matrices of the *B*-axis, *P*_w_ is the initial coordinates of point *P* in the coordinate system *O*_2_-*X*_3_*Y*_3_*Z*_3_, which can be denoted by

(19) $$P_{w}=\left[\begin{array}{c} R_{w}\sin\phi \\ -R_{w}\cos\phi \\ 0\\ 1 \end{array}\right]$$

Referring to Equations (3) and (4), *R*_B_ can be expressed by

(20) $$R_{B}=\left[\begin{array}{cccc} \cos\left(\alpha +\varepsilon_{\alpha }\right) & -\varepsilon_{B}{}_{z} & \sin\left(\alpha +\varepsilon_{\alpha }\right) & \delta_{B}{}_{x}\\ \varepsilon_{B}{}_{z}\cos\left(\alpha +\varepsilon_{\alpha }\right)+\varepsilon_{B}{}_{x}\sin\left(\alpha +\varepsilon_{\alpha }\right) & 1 & \varepsilon_{B}{}_{z}\sin\left(\alpha +\varepsilon_{\alpha }\right)-\varepsilon_{B}{}_{x}\cos\left(\alpha +\varepsilon_{\alpha }\right) & m+\delta_{By}\\ \varepsilon_{B}{}_{x}\varepsilon_{B}{}_{z}\cos\left(\alpha +\varepsilon_{\alpha }\right)-\sin\left(\alpha +\varepsilon_{\alpha }\right) & \varepsilon_{B}{}_{x} & \varepsilon_{B}{}_{x}\varepsilon_{B}{}_{z}\sin\left(\alpha +\varepsilon_{\alpha }\right)+\cos\left(\alpha +\varepsilon_{\alpha }\right) & -h+\delta_{B}{}_{z}\\ 0 & 0 & 0 & 1 \end{array}\right]$$

Referring to Equation (5), *T*_Z_ can be expressed by

(21) $$T_{Z}=\left[\begin{array}{cccc} 1 & -\varepsilon_{Z}{}_{z} & \varepsilon_{Zy} & e+\delta_{\mathrm{Zx}}\\ \varepsilon_{Z}{}_{z} & 1 & -\varepsilon_{Z}{}_{x} & d+\delta_{Zy}\\ -\varepsilon_{Zy} & \varepsilon_{Z}{}_{x} & 1 & n+\delta_{z}\\ 0 & 0 & 0 & 1 \end{array}\right]$$

Similarly, *T*_X_ can be expressed by

(22) $$T_{X}=\left[\begin{array}{cccc} 1 & -\varepsilon_{X}{}_{z} & \varepsilon_{Xy} & l+\delta_{x}\\ \varepsilon_{X}{}_{z} & 1 & -\varepsilon_{X}{}_{x} & -g-i+\delta_{Xy}\\ -\varepsilon_{Xy} & \varepsilon_{X}{}_{x} & 1 & f+\delta_{Xz}\\ 0 & 0 & 0 & 1 \end{array}\right]$$

According to Equation (8), *R*_C_ can be expressed by

(23) $$R_{C}=\left[\begin{array}{cccc} \cos\left(\theta +\varepsilon_{\theta }\right) & -\sin\left(\theta +\varepsilon_{\theta }\right) & \varepsilon_{C}{}_{y} & \delta_{C}{}_{x}\\ \sin\left(\theta +\varepsilon_{\theta }\right) & \cos\left(\theta +\varepsilon_{\theta }\right) & -\varepsilon_{C}{}_{x} & \delta_{Cy}\\ \varepsilon_{C}{}_{x}\sin\left(\theta +\varepsilon_{\theta }\right)-\varepsilon_{C}{}_{y}\cos\left(\theta +\varepsilon_{\theta }\right) & \varepsilon_{C}{}_{x}\cos\left(\theta +\varepsilon_{\theta }\right)+\varepsilon_{C}{}_{y}\left(\theta +\varepsilon_{\theta }\right) & 1 & -j+\delta_{C}{}_{z}\\ 0 & 0 & 0 & 1 \end{array}\right]$$

Regarding positioning error as a part of the geometric error and taking all positioning errors of the four axes into consideration, the generated wavy face form deviation can be expressed by

(24) $$\{\begin{array}{l} \begin{array}{cc} x= & u(R_{w},d,e,f,g,i,j,l^{\prime },m,n^{\prime },h,\alpha^{\prime },\theta^{\prime },\phi ,\varepsilon_{B}{}_{x},\varepsilon_{B}{}_{z},\delta_{B}{}_{x},\delta_{By},\delta_{B}{}_{z},\varepsilon_{Z}{}_{x},\varepsilon_{Zy},\varepsilon_{Z}{}_{z},\delta_{\mathrm{Zx}},\delta_{Zy},\varepsilon_{X}{}_{x},\varepsilon_{Xy},\\ & \varepsilon_{X}{}_{z},\delta_{Xy},\delta_{Xz},\varepsilon_{C}{}_{x},\varepsilon_{C}{}_{y},\delta_{C}{}_{x},\delta_{Cy},\delta_{C}{}_{z}) \end{array}\\ \begin{array}{cc} y= & v(R_{w},d,e,f,g,i,j,l^{\prime },m,n^{\prime },h,\alpha^{\prime },\theta^{\prime },\phi ,\varepsilon_{B}{}_{x},\varepsilon_{B}{}_{z},\delta_{B}{}_{x},\delta_{By},\delta_{B}{}_{z},\varepsilon_{Z}{}_{x},\varepsilon_{Zy},\varepsilon_{Z}{}_{z},\delta_{\mathrm{Zx}},\delta_{Zy},\varepsilon_{X}{}_{x},\varepsilon_{Xy},\\ & \varepsilon_{X}{}_{z},\delta_{Xy},\delta_{Xz},\varepsilon_{C}{}_{x},\varepsilon_{C}{}_{y},\delta_{C}{}_{x},\delta_{Cy},\delta_{C}{}_{z}) \end{array}\\ \begin{array}{cc} z= & w(R_{w},d,e,f,g,i,j,l^{\prime },m,n^{\prime },h,\alpha^{\prime },\theta^{\prime },\phi ,\varepsilon_{B}{}_{x},\varepsilon_{B}{}_{z},\delta_{B}{}_{x},\delta_{By},\delta_{B}{}_{z},\varepsilon_{Z}{}_{x},\varepsilon_{Zy},\varepsilon_{Z}{}_{z},\delta_{\mathrm{Zx}},\delta_{Zy},\varepsilon_{X}{}_{x},\varepsilon_{Xy},\\ & \varepsilon_{X}{}_{z},\delta_{Xy},\delta_{Xz},\varepsilon_{C}{}_{x},\varepsilon_{C}{}_{y},\delta_{C}{}_{x},\delta_{Cy},\delta_{C}{}_{z})-\gamma_{0}\left(\sqrt{x^{2}+y^{2}}-R_{2}\right)\left(\cos\left(N\arctan\frac{y}{x}\right)-1\right) \end{array} \end{array}$$

**Figure 7:**
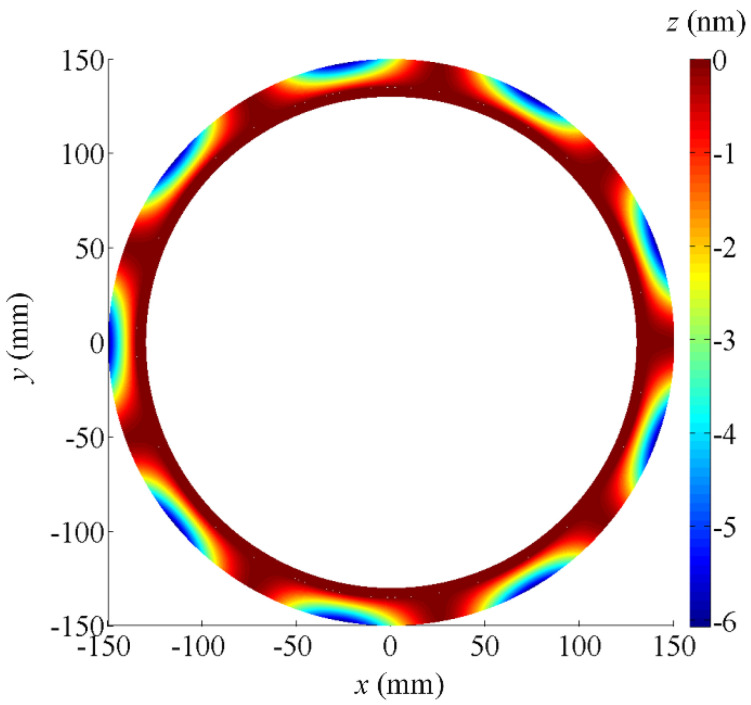
Generated WTD seal shape that concerns geometric errors.

**Figure 8:**
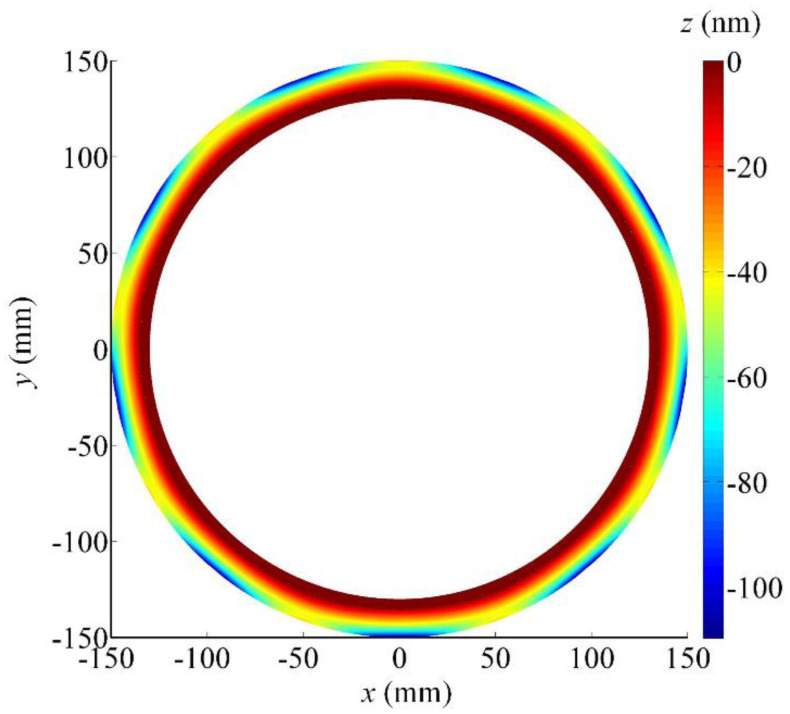
Form deviations map of the ground wavy face concerning geometric errors.

AMETEK^®^ Precitech, Inc. (Keene, NH, USA) and Moore Tool Company, Inc. (Bridgeport, CT, USA) are two famous companies that are producing ultra-precision machine tools and their manufacturing abilities represent the top-level of advanced manufacturing techniques. Being similar cases, relevant parameters can refer to the Nanotech 450UPL (Moore Tool Company, Inc., Bridgeport, CT, USA), the Nanoform^®^ 700 ultra (AMETEK® Precitech, Inc., Keene, NH, USA), and the Nanoform^®^ L 1000 (AMETEK® Precitech, Inc., Keene, NH, USA) machine tools. Linear axes are oil hydrostatic slideways whose position feedback resolution reaches 0.03 nm and the straightness of the horizontal/vertical is less than 0.5 μm over 350 mm full stroke, roll, yaw and pitch motion is 2 arcsec. Rotary axes are oil hydrostatic or air bearing spindles whose radial/axial error motion is less than 0.1 μm and positioning accuracy is ±1 arcsec. Significantly, since the effective stroke of the *X*-axis, *Z*-axis and *B*-axis are all extremely small, the errors of straightness, squareness, and strokes are all ignored. Taking other parameters as variables to calculate the form error of the ground wavy face—in reference to Equation (24)—the results are shown in Figure 7 and Figure 8. The maximal amplitude of the ground wavy face is about 6.07 μm which is larger than the design value and the form error is 109.74 nm which is limited to the length of one helium light band.

## 7. Discussion

Referring to the calculated results above, it is found that the *Z*-axis and *B*-axis are located in sensitive directions of the processing error while the *X*-axis and *C*-axis are located in insensitive directions. In particular, the positioning error of the *Z*-axis has no influence on the ground wavy face in principle, except for the axial location of the wavy face and seal dam, but its accuracy degree still should be high enough. Otherwise, the offset of the boundary between the wavy face and seal dam will evidently cause the chances of total form error of the WTD seal to increase. By carefully observing Figure 2, Figure 3 and Figure 4, it is obvious that the ground form error of the wavy face is mainly affected by the motional accuracy of the *B*-axis. When a positioning error of the *B*-axis is 2 or −2 arcsec, the form error of the ground wavy face will exceed 232 nm. However, there should be a certain margin of error caused by other factors such as kinematic errors, thermally induced errors, cutting-force-induced errors, fixture-dependent errors, assembly error, servo tracking error, etc [30,31]. As a positioning error is limited to within ±1 arcsec, which is almost the limit of fabricating such a large rotary table by modern manufacturing techniques, the *B*-axis will be the most important component of the four-axis grinder. In comparison, the *X*-axis and *C*-axis can be chosen to a low degree of accuracy because their motions are in the circumferential direction of the wavy face.

In general, it is unacceptable to increase the machining accuracy by reducing geometric errors regardless of the component costs [32]. Therefore, the determination of an optimal balance—not only between total cost and accuracy of machine tool but also the accuracy matching-relationship among different components—is a critical problem that needs to be of high concern. If a cost budget is allowed, it would be better that the accuracy of the *X*-axis and *C*-axis are close to that of the *Z*-axis and *B*-axis, respectively. Especially since the main function of the *X*-axis is to determine the wavy dam position according to the seal size. Hence, it can be removed if it is just used to fabricate specific size wavy seals in order to improve the accuracy of the ultraprecision grinder.

In the present paper, only key geometric errors are discussed and some of the minor errors are ignored. However, those errors should also be recognized and eliminated as much as possible. For instance, machining such seals with extremely high accuracy must be executed in clean rooms, so the temperature can be controlled precisely so that the thermally induced errors can be restricted. Furthermore, other means also can be considered: such as using a pneumatic isolation system to restrain vibration with an optional adaptive control technology to improve the servo resolution, utilizing precision machining technology to fabricate high precision accessories and decrease fixture-dependent errors, adopting micro-feeding technology to reduce cutting-force-induced errors, etc. Overall, constructing the proposed four-axis grinder will pose a challenge to modern machining techniques, but it is practicable.

## 8. Conclusions

1. The *Z*-axis and *B*-axis are located in sensitive directions of the processing error while the *X*-axis and *C*-axis are located in insensitive directions. The positioning accuracy of the *X*-axis, *Z*-axis, *B*-axis and *C*-axis components is recommended to be within ±10 μm, ±0.1 μm, ±1 arcsec and ±60 arcsec, respectively.
2. The ground form error of the wavy face is mainly affected by the positioning accuracy of the *B*-axis which can increase the form deviations, apparently. Therefore, the accuracy of the *B*-axis should be limited as low as the manufacturers can provide.
3. Taking the geometric errors of all the four axes into consideration, the maximal amplitude of the ground wavy face is about 6.07 μm which is a little larger than the design value and the form error is only 109.74 nm which is far less than the allowable value of 580 nm.
4. All the machine components with assumed geometric errors can be fabricated by modern manufacturing techniques and the calculated results can serve as a theoretical basis for the design and development of the four-axis grinder.

## Figures and Tables

**Table 1:** Effects of assumed positioning error of transitional axes on form error of the ground wavy face.

Axis Name	Positioning Error (μm)	Form Error (nm)
*X*-axis	2	77.18
	−2	77.17
	5	77.20
	−5	77.19
	10	77.29
	−10	77.26
	20	77.62
	−20	77.55
*Z*-axis	-	57.88

**Table 2:** Effects of assumed positioning error of rotational axes on form error of the ground wavy face.

Axis Name	Positioning Error (arcsec)	Form Error (nm)
*B*-axis	0.1	77.17
	−0.1	77.17
	0.5	90.88
	−0.5	90.77
	1	136.09
	−1	135.90
	2	232.80
	−2	232.54
*C*-axis	10	80.66
	−10	73.68
	30	87.64
	−30	66.70
	60	98.11
	−60	56.23
	120	119.06
	−120	35.28
